# Effects of a New Eyelid Shampoo on Lid Hygiene and Eyelash Length in Patients with Meibomian Gland Dysfunction: A Comparative Open Study

**DOI:** 10.1155/2016/4292570

**Published:** 2016-12-19

**Authors:** Asuka Kobayashi, Takeshi Ide, Teruki Fukumoto, Emiko Miki, Kazuo Tsubota, Ikuko Toda

**Affiliations:** ^1^Minamiaoyama Eye Clinic, Renai Aoyama Building 4F, 3-3-11 Kitaaoyama, Minato-ku, Tokyo 107-0061, Japan; ^2^Department of Ophthalmology, Keio University School of Medicine, 35 Shinanomachi, Shinjuku-ku, Tokyo 1608582, Japan; ^3^Kikugawa Eye Clinic, 5134 Kamo Kikukawa-shi, Shizuoka, Japan; ^4^Miki Eye Clinic, 1-5 Iwata Tsu-shi, Mie, Japan

## Abstract

*Purpose*. Meibomian gland dysfunction (MGD) can lead to abnormalities in the composition and function of tear film, resulting in dry eye. Eyelid hygiene is a key to management of MGD. We tested a novel eyelid shampoo (Eye Shampoo Long, ESL) for its ability to maintain lid hygiene. This shampoo is nonirritating and can potentially lengthen eyelashes. This study was aimed to evaluate the efficacy of ESL in the treatment of MGD and its effects on eyelash length.* Methods*. Ten patients with MGD and 10 healthy subjects without MGD applied ESL twice daily for 8 weeks. Patients were examined for lid margin and dry eye before and after the trial. Subjective symptoms were evaluated. Eyelash length was measured at baseline and at the end of the trial.* Results*. In the MGD group, significant improvements were observed in subjective symptoms obstruction of the meibomian orifice, secretion of meibum, eyelashes contamination, eyelid margin foam, and SPK. Eyelash length became significantly longer.* Conclusions*. Maintaining eyelid hygiene using ESL improved the eyelid margins and symptoms of dry eye in MGD patients and increased eyelash length. These findings are promising and warrant confirmation in a larger randomized controlled study.

## 1. Background

Meibomian gland dysfunction (MGD) is a chronic, diffuse abnormality of the meibomian glands. It is commonly characterized by obstruction of the terminal ducts and qualitative and/or quantitative changes in glandular secretions [1]. These can cause changes in tear film, eye irritation, clinically apparent inflammation, and diseases of the ocular surface [2].

MGD can be divided into low-delivery and high-delivery states based on meibomian gland secretions. Obstructive MGD is the most common form of dry eye and is categorized as a low-delivery state [2]. It is also a major cause of evaporative dry eye disease. In this condition, decreased delivery of meibum to the ocular surface causes increased tear evaporation. The resulting increase in tear film osmolality leads to ocular surface damage [3].

Eyelid hygiene is essential in the management of MGD and plays a fundamental role in decreasing the load of lipolytic bacteria and the amount of potentially toxic lipids on the ocular surface originating from products that cause tear film spoilage (e.g., free fatty acids) [4–8]. Several lid scrubs are commercially available and have been shown to be effective for managing dry eye disease [7–11]. Baby shampoo is one such lid scrub and for many physicians is the treatment of choice for chronic blepharitis. However, even though it is gentle and pH-balanced, ocular surface irritation can still occur when this conventional shampoo contacts the ocular surface.

In this study, we investigated a novel eyelid shampoo formulated for lid hygiene regimens. This shampoo is nonirritating and potentially lengthens the eyelashes. The purpose of this study was to evaluate the effects of this newly developed eye-cleansing formulation on MGD and eyelash length.

## 2. Material and Methods

The study protocol was prospectively reviewed and approved by the Minamiaoyama Eye Clinic Internal Review Board and was conducted according to the tenets of the Declaration of Helsinki. All subjects provided written informed consent to participate after receiving a detailed explanation of the study.

### 2.1. Subjects

Ten patients (2 men, 8 women) with dry eye associated with MGD were enrolled into the study. The patients in the MGD-related dry eye group were diagnosed according to the 2006 guidelines published by Shimazaki [12]. To determine whether the trial shampoo (Eye Shampoo Long [ESL], MediProduct Co., Ltd., Tokyo, Japan) had an effect on eyelash growth, we included 10 healthy subjects (4 men, 6 women) for measurement of eyelash length.

### 2.2. Eyelid Hygiene Product

**Figure 1:**
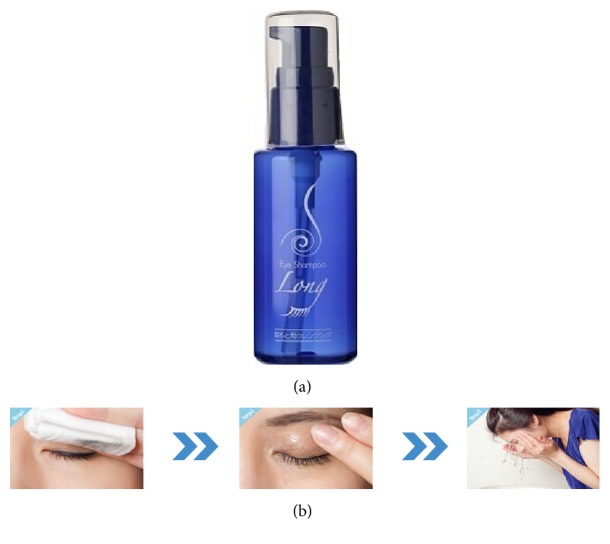
Lid hygiene technique. (a) ESL. (b) Lid cleansing using ESL. ESL: Eye Shampoo Long.

ESL is a nonirritating, eye-cleansing formulation (Figure 1(a)) with a pH of 7.4 and an osmolality of 300 mOsm/L, which is similar to that of normal tear film [13]. The product contains components that are anti-inflammatory (dipotassium glycyrrhizate, cholecalciferol [vitamin D3]) and moisturizing (blends of ceramide, Kjellmaniella gyrate extract) and promote hair growth (cholecalciferol [vitamin D3], Panax ginseng root extract, Kjellmaniella gyrate extract, and amino acids (see Table 1 )) [14–18]. The manufacturer confirmed product safety.

All participants used ESL twice a day for 8 weeks according to the prescribed study washing regimen, which entailed pumping the shampoo onto one hand or a piece of cotton and spreading it gently around the eyes. Next, the study participants lightly massaged their eyelids to remove impurities located at the eyelash roots and rinsed their eyes with water (Figure 1(b)). During the study, subjects were asked to continue any eye drops that they were using at enrollment.

### 2.3. Study Tests and Examinations

Examinations were performed in 10 patients with MGD-associated dry eye and in 10 healthy subjects to check for a possible effect on eyelash growth. The characteristics of the two groups are presented in Table 2. All subjects completed a background survey (Table 3) at the time of enrollment (baseline) and at the end of the 8-week treatment period.

The examinations (Table 4) included tear breakup time, fluorescein staining score, and lipid layer thickness (LipiView, TearScience, Inc., Morrisville, NC, USA). Eyelid margin findings (meibomian gland blockage, eyelash contamination, eyelid margin foam, and decreased meibum secretion) were graded on a 5-point scale ranging from 0 to 4 (Table 5). The change in meibum secretion was evaluated by extruding meibum from the lower eyelid using MGE-100 (TearScience, Inc.). The severity of superficial punctate keratopathy (SPK) was evaluated using a 4-point scale ranging from 0 to 3. The corneal surface was divided into nasal, temporal, and central regions, and SPK was graded in each area. The final SPK score was calculated as the sum of all regional scores. Visual acuity, intraocular pressure (IOP), and refractive error were measured at each study visit. The longest eyelash on the left eye was identified, pulled, and taped to a piece of paper for measurement.

Dry eye symptoms were evaluated using a questionnaire administered at baseline and after 8 weeks of using ESL. The questionnaire assessed 17 ocular symptoms (Table 6). Responses of “none,” “mild,” “moderate,” and “severe” were scored as 0, 1, 2, and 3, respectively.

### 2.4. Statistical Analyses

The data are presented as the mean ± standard deviation. Differences in subjective symptoms and findings at the eyelid margin (meibomian gland blockage, eyelash contamination, lid margin foam, and decreased meibum secretion) were tested for statistical significance using the Mann—Whitney *U* test. Differences in ocular surface findings (including SPK) and eyelash length were tested for statistical significance using the Wilcoxon signed rank test. Statistical significance was defined as *p* < .05.

## 3. Results

### 3.1. Subjective Symptoms

**Figure 2:**
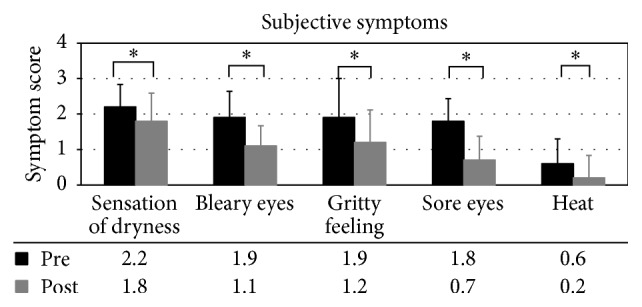
Subjective symptom scores for patients with meibomian gland dysfunction.

The patients with MGD showed a significant improvement from baseline in subjective symptoms after 8 weeks using ESL. Sensation of dryness, bleary eyes (foreign body sensation), grittiness (foreign body sensation), sore eyes (burning sensation), and heat (burning sensation) improved significantly (*p* < .05) during this time (Table 7, Figure 2).

### 3.2. Findings at the Eyelid Margin

**Figure 3:**
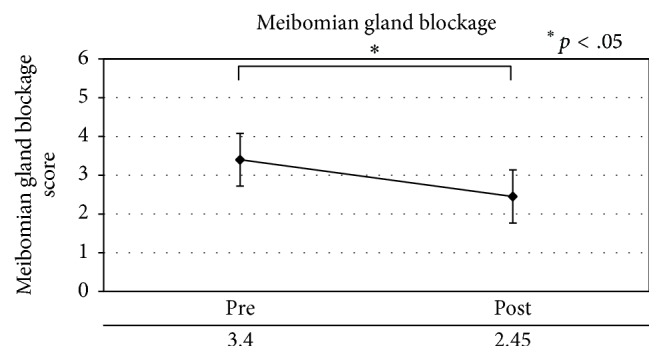
Meibomian gland blockage in a patient with meibomian gland dysfunction.

**Figure 4 fig4:**
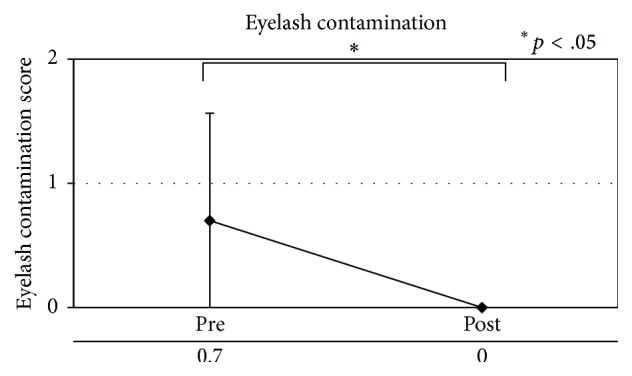
Eyelash contamination in patients with meibomian gland dysfunction.

**Figure 5 fig5:**
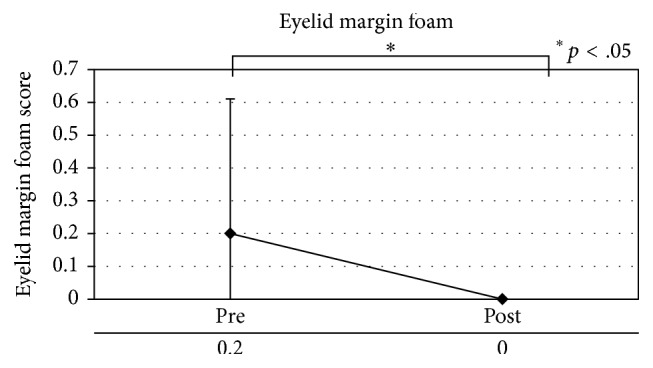
Eyelid margin foam in patients with meibomian gland dysfunction.

**Figure 6:**
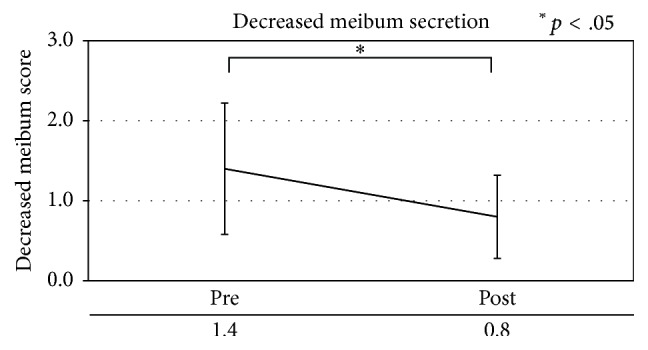
Decreased meibum secretion in patients with meibomian gland dysfunction.

Patients with MGD showed a significant improvement (*p* < .05) in meibomian gland blockage, eyelash contamination, and eyelid margin foam, as well as decreased meibum secretion (Table 7, Figures 3 [Fig fig4] [Fig fig5]–6).

### 3.3. Eyelash Growth

**Figure 7:**
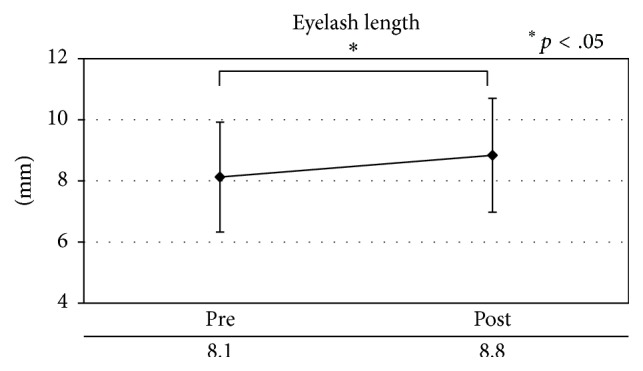
Eyelash length.

Eyelashes were significantly longer after 8 weeks of using ESL than they were at baseline in the patients with MGD and in the healthy subjects (*p* < .05; Table 7, Figure 7).

### 3.4. Ocular Surface and Tear Function

**Figure 8:**
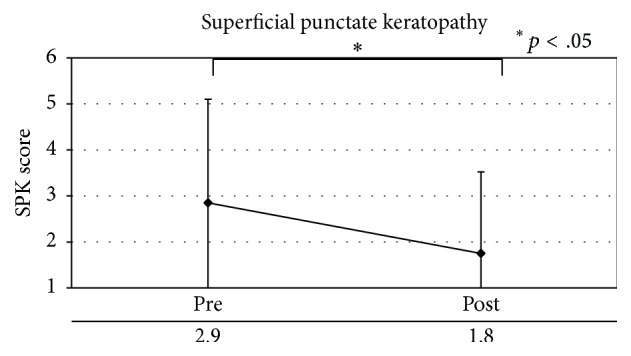
Superficial punctate keratopathy in patients with meibomian gland dysfunction.

SPK significantly improved in the MGD group (*p* < .05) during the 8-week study period. However, no significant changes were observed in tear breakup time or lipid layer thickness this time (Table 7, Figure 8).

## 4. Discussion

Our results indicate that the condition of the eyelids improved markedly after using ESL for 8 weeks. The objective findings for MGD and the ocular surface improved, subjective dry eye symptoms (e.g., chronic ocular discomfort) lessened, and eyelash length increased.

It was suspected that ESL would not be effective because a mild shampoo is likely to have limited cleansing ability. However, we found improvements in the cleanliness of the eyelashes and meibomian blockage.

In general, lid hygiene regimens consist of application of warm compresses and lid massage to clear meibomian gland blockage. Antibiotics and anti-inflammatory agents can also be used to improve the quality of the meibum. Tear substitutes and additional lubricants can help to alleviate the symptoms of dry eye. Topical antibiotic ointments may be used in moderate and severe cases. However, the treatments available have somewhat variable effects and must be continued over long periods to achieve a satisfactory reduction of symptoms. As a result, patients often discontinue treatment [6, 7].

The patients in this study were compliant with the lid hygiene regimen partly because of improvements in their MGD symptoms, such as eyelash contamination, and subjective symptoms. ESL is believed to improve compliance because the regimen is less complicated than that required when using conventional baby shampoo regimens; for example, unlike baby shampoo, this product does not need to be diluted. No treatments for MGD that are effective in long-term use have been described in the literature. However, ESL is easy to handle and reduces irritation; therefore patients using it may be more likely to continue their treatment.

The eyelash-lengthening effect of ESL maybe due to its vitamin D and hair growth-promoting components and/or the effects of better hygiene [14, 15]. It is possible that lengthening of the eyelashes occurred once the eyelashes were cleaned by washing and the condition of the eyelash roots improved. Therefore, future studies should compare ESL with other formulations to investigate this effect. The mRNA encoding 25-hydroxylase, the vitamin D activation enzyme, was recently shown to be present in some ocular cells [16]. However, expression of this gene in the meibomian glands of epithelial cells has not been confirmed. It is possible that vitamin D_3_ is activated in the meibomian gland epithelium and induces eyelash growth, as has been demonstrated on the scalp [14–16, 19]. Future studies should be performed to determine whether the vitamin D activation enzyme is expressed in the epithelium of the meibomian glands. Lastly, lid massage may have promoted eyelash growth because of the presence of Panax ginseng extract and Kjellmaniella gyrate extract, both of which are known to stimulate hair growth [17, 18].

The effect of ESL on lid hygiene was similar to that of already available commercial products [4]. However, ESL is less irritating and has the additional benefit of lengthening the eyelashes. The lash-lengthening effect of ESL may further improve compliance with the lid hygiene regimen and encourage patients with MGD to wash their eyelids regularly, just as they brush their teeth. ESL can also be used as a make-up remover, so it can reduce MGD caused by residual eye make-up.

In the present study, no adverse events such as redness, discharge, pupillary formation of congestive, and edema were observed and good results were obtained using ESL. Moreover, no participants reported irritation using ESL. Previous report admitted product safety [4]. In addition, participants reported that ESL was easily available and that they felt reduced MGD symptoms and 30% of the subjects continue to use the ESL. These suggest that continued use of ESL could have additional favorable effects on the meibomian gland; however, we have to admit that the sample size is small. Further investigation is warranted [4].

In summary, ESL is the first product in Japan which can take care of eyelashes as well as MGD treatment. ESL effectively improved the signs and symptoms of MGD and lengthened eyelashes, which may encourage patients to continue using the product. Both of these factors would encourage compliance with a lid hygiene regimen.

## Figures and Tables

**Table 1:** Composition of Eye Shampoo Long.

	INCI code	Function
1	Water	Base
2	Butylene glycol	Moisturizer
Content less than 1%		
3	Alanine	Moisturizer
4	Allantoin	Moisturizer
5	Arginine	pH adjuster
6	Carbomer	Thickener
7	Ceramide AP	Emollient
8	Ceramide EOP	Emollient
9	Ceramide NP	Emollient
10	Cholecalciferol	Emollient
11	Cholesterol	Emollient
12	Citric acid	pH adjuster
13	Dipotassium glycyrrhizinate	Skin-conditioning agent
14	Disodium guanylate	Moisturizer
15	Disodium inosinate	Moisturizer
16	Glutamic acid	Moisturizer
17	Glycine	Moisturizer
18	Histidine HCl	Moisturizer
19	Isoleucine	Moisturizer
20	Kjellmaniellagyrata extract	Moisturizer
21	Leucine	Moisturizer
22	Lysine HCl	Moisturizer
23	Panax ginseng root extract	Moisturizer
24	PEG-25 glyceryl isostearate	Detergent
25	Phenoxyethanol	Preservative
26	Phenylalanine	Moisturizer
27	Phytic acid	Chelating agent
28	Phytosphingosine	Emollient
29	Proline	Moisturizer
30	Sericin	Moisturizer
31	Serine	Moisturizer
32	Silver oxide	Preservative
33	Sodium aspartate	Moisturizer
34	Sodium chloride	Base
35	Sodium lauroyl lactylate	Moisturizer
36	Sodium polyacrylate	Thickener
37	Taurine	Moisturizer
38	Threonine	Moisturizer
39	Tyrosine	Moisturizer
40	Valine	Moisturizer
41	Xanthan gum	Thickener
42	Zea mays (corn) oil	Emollient

**Table 2:** Demographic characteristics of study participants.

Parameter	MGD patients (*n* = 10)	Controls, eyelash length (*n* = 10)
Male	2	4
Female	8	6
Age (years)	53.8 (32–79)	34.0 (34–54)

**Table 3:** Questionnaire of eye care habits.

	Yes	No	Occasionally	Hardly ever
Use of contact lens				
Anamnesis of LASIK				
Regular eye washing				
Anamnesis of stye and chalazion				
Use of eye drops				

**Table 4:** Specialized and nonspecialized tests for meibomian gland dysfunction, dry eye, and eyelash length.

Category	Test
Symptoms	Original questionnaires^*∗*^
Signs	
Meibomian function	Eye lash contamination
	Meibomian gland blockage
	Eyelid margin foam
	Decreased meibum secretion
Ocular surface	Tear breakup time
	Superficial punctate keratopathy
	Lipid layer thickness
Eyelash	Length

^*∗*^See Table 5 for grading scales.

**Table 5:** Grading scales for signs of meibomian gland dysfunction.

*Eyelash contamination*	
0	Clear
1	Slight contamination
2	Mild
3	Moderate
4	Severe

*Meibomian orifice obstruction*	
0	Clear liquid
1	Yellowish liquid
2	Opaque and toothpaste-like consistency
3	Thickened white material
4	Not possible

*Meibomian gland blockage *	
0	None
1	<25% blocked glands
2	25% to <50%
3	50% to <75%
4	75% or more

*Lid margin foam*	
0	Clear
1	At the canthus
2	Lower or upper
3	Lower and upper
4	Lower and upper or at the canthus

**Table 6:** Results of ocular symptoms questionnaire before and after 8 weeks of the study intervention.

	0 (none)	1 (mild)	2 (moderate)	3 (severe)
(1) Eye discomfort (vague discomfort in eyes)				
(2) Sensations of dryness				
(3) Bleary eyes (foreign body sensation)				
(4) Grittiness (foreign body sensation)				
(5) Sore eyes (burning sensation)				
(6) Heat (burning sensation)				
(7) Eye discharge (gum in eye)				
(8) Stringy mucus				
(9) Blurriness				
(10) Eye strain				
(11) Eye pain				
(12) Lacrimation (excessive watering of the eye)				
(13) Itchiness				
(14) Red eye				
(15) Heaviness				
(16) Extra sensitivity to light				
(17) Excessive frequent eye blinking (frequent blinks)				

**Table 7:** Comparison of symptoms, meibomian gland function, eyelash length, and ocular surface according to treatment group (we listed the items with *p* < .05 only).

Group/task		Baseline (mean ± SD)	8 weeks (mean ± SD)	*p* versus baseline
*Subjective symptoms*				
	Sensation of dryness	2.20 ± 0.63	1.80 ± 0.79	.046
	Bleary eyes (foreign body sensation)	1.90 ± 0.74	1.10 ± 0.57	.011
	Gritty feeling (foreign body sensation)	1.90 ± 1.10	1.20 ± 0.92	.020
	Sore eyes (burning sensation)	1.80 ± 0.63	0.70 ± 0.67	.009
	Heat (burning sensation)	0.60 ± 0.70	0.20 ± 0.63	.046

*Meibomian gland function*				
	Eyelash contamination	0.70 ± 0.86	0.00 ± 0.00	.004
	Meibomian gland blockage	3.40 ± 0.68	2.45 ± 0.69	.000
	Eyelid margin foam	0.20 ± 0.41	0.00 ± 0.00	.046
	Decreased meibum secretion	1.40 ± 0.82	0.80 ± 0.52	.038

*Eyelash length*				
MGD patients and healthy subjects	Length	8.13 ± 1.80	8.84 ± 1.86	.020

*Ocular surface*				
	Superficial punctate keratopathy	2.95 ± 2.25	1.75 ± 1.77	.002

MGD: meibomian gland dysfunction; SD: standard deviation.

## Abbreviations

MGD: Meibomian gland dysfunction
ESL: Eye Shampoo Long
SPK: Superficial punctate keratopathy
IOP: Intraocular pressure.

## References

[1] Nichols K. K., Foulks G. N., Bron A. J. (2011). The International Workshop on meibomian gland dysfunction: executive summary. *Investigative Ophthalmology and Visual Science*.

[2] Nelson J. D., Shimazaki J., Benitez-del-Castillo J. M. (2011). The International Workshop on meibomian gland dysfunction: report of the definition and classification subcommittee. *Investigative Ophthalmology & Visual Science*.

[3] Yokoi N., Mossa F., Tiffany J. M., Bron A. J. (1999). Assessment of meibomian gland function in dry eye using meibometry. *Archives of Ophthalmology*.

[4] Okura M., Kawashima M., Katagiri M., Shirasawa T., Tsubota K. (2015). New eye cleansing product improves makeup-related ocular problems. *Journal of Ophthalmology*.

[5] Polack F. M., Goodman D. F. (1988). Experience with a new detergent lid scrub in the management of chronic blepharitis. *Archives of Ophthalmology*.

[6] Paugh J. R., Knapp L. L., Martinson J. R., Hom M. M. (1990). Meibomian therapy in problematic contact lens wear. *Optometry and Vision Science*.

[7] Romero J. M., Biser S. A., Perry H. D. (2004). Conservative treatment of meibomian gland dysfunction. *Eye and Contact Lens*.

[8] Key J. E. (1996). A comparative study of eyelid cleaning regimens in chronic blepharitis. *CLAO Journal*.

[9] Bowman R. W., Dougherty J. M., McCulley J. P. (1987). Chronic blepharitis and dry eyes. *International Ophthalmology Clinics*.

[10] Wittpenn J. R. (1995). EyeScrub: simplifying the management of blepharitis. *Journal of Ophthalmic Nursing & Technology*.

[11] Goto E., Shimazaki J. (1997). Meibomian gland dysfunction and its treatment. *Atarashii Ganka*.

[12] Shimazaki J. (2006). Definition and diagnosis of dry eye. *Atarashii Ganka*.

[13] Johnson M. E., Murphy P. J., Boulton M. (2006). Effectiveness of sodium hyaluronate eyedrops in the treatment of dry eye. *Graefe's Archive for Clinical and Experimental Ophthalmology*.

[14] Vegesna V., O'Kelly J., Uskokovic M. (2002). Vitamin D3 analogs stimulate hair growth in nude mice. *Endocrinology*.

[15] Amor K. T., Rashid R. M., Mirmirani P. (2010). Does D matter? The role of vitamin D in hair disorders and hair follicle cycling. *Dermatology Online Journal*.

[16] Alsalem J. A., Patel D., Susarla R. (2014). Characterization of vitamin D production by human ocular barrier cells. *Investigative Ophthalmology and Visual Science*.

[17] Matsuda H., Yamazaki M., Asanuma Y., Kubo M. (2003). Promotion of hair growth by Ginseng Radix on cultured mouse vibrissal hair follicles. *Phytotherapy Research*.

[18] Sawako H., Sakai N., Takahashi K. (2012). Efficacy of the hair restorer containing fucoidan from Gagome kombu in women with diffuse hair loss. *Fragrance Journal*.

[19] Soga H., Yokoyama Y., Gotou A. (2012). Changes of the hair property and the scalp condition by the chronic treatment of the scalp massage. *Fragrance Journal*.

